# Rare microbial taxa as the major drivers of nutrient acquisition under moss biocrusts in karst area

**DOI:** 10.3389/fmicb.2024.1384367

**Published:** 2024-05-01

**Authors:** Xintong Dong, Man Chen, Qi Chen, Kangfei Liu, Jie Long, Yunzhou Li, Yinuo Ren, Tao Yang, Jinxing Zhou, Saman Herath, Xiawei Peng

**Affiliations:** ^1^College of Biological Sciences and Technology, Beijing Forestry University, Beijing, China; ^2^Jianshui Research Station, School of Soil and Water Conservation, Beijing Forestry University, Beijing, China; ^3^Department of Export Agriculture, Faculty of Animal Science and Export Agriculture, Uva Wellassa University, Badulla, Sri Lanka; ^4^Beijing Key Laboratory of Food Processing and Safety in Forestry, Beijing Forestry University, Beijing, China; ^5^National Engineering Laboratory for Tree Breeding, Beijing Forestry University, Beijing, China

**Keywords:** land degradation, bryophytes, extracellular enzyme stoichiometry, assembly processes, co-occurrence networks

## Abstract

Karst rocky desertification refers to the process of land degradation caused by various factors such as climate change and human activities including deforestation and agriculture on a fragile karst substrate. Nutrient limitation is common in karst areas. Moss crust grows widely in karst areas. The microorganisms associated with bryophytes are vital to maintaining ecological functions, including climate regulation and nutrient circulation. The synergistic effect of moss crusts and microorganisms may hold great potential for restoring degraded karst ecosystems. However, our understanding of the responses of microbial communities, especially abundant and rare taxa, to nutrient limitations and acquisition in the presence of moss crusts is limited. Different moss habitats exhibit varying patterns of nutrient availability, which also affect microbial diversity and composition. Therefore, in this study, we investigated three habitats of mosses: autochthonal bryophytes under forest, lithophytic bryophytes under forest and on cliff rock. We measured soil physicochemical properties and enzymatic activities. We conducted high-throughput sequencing and analysis of soil microorganisms. Our finding revealed that autochthonal moss crusts under forest had higher nutrient availability and a higher proportion of copiotrophic microbial communities compared to lithophytic moss crusts under forest or on cliff rock. However, enzyme activities were lower in autochthonal moss crusts under forest. Additionally, rare taxa exhibited distinct structures in all three habitats. Analysis of co-occurrence network showed that rare taxa had a relatively high proportion in the main modules. Furthermore, we found that both abundant and rare taxa were primarily assembled by stochastic processes. Soil properties significantly affected the community assembly of the rare taxa, indirectly affecting microbial diversity and complexity and finally nutrient acquisition. These findings highlight the importance of rare taxa under moss crusts for nutrient acquisition. Addressing this knowledge gap is essential for guiding ongoing ecological restoration projects in karst rocky desertification regions.

## 1 Introduction

Land degradation associated with karst rocky desertification is caused by both natural processes and human activities on a fragile karst background (Meng et al., 2021). Rocky desertification has become a challenge for the sustainable ecological development of Southwest China (Yang et al., 2022a). Moss biocrusts are crucial for reducing and preventing soil erosion on rock surfaces and for supporting the long-term viability of the vegetation restoration process (Kidron and Drahorad, 2022). On the other hand, the microbial community in these moss biocrusts plays a significant ecological role (Cheng et al., 2022). Therefore, it is essential to comprehend the functions and mechanisms of these microorganisms in ecological functions.

Moss biocrusts in the karst areas contain a large number of highly diverse microorganisms. Microbial diversity is a fundamental aspect of supporting the services provided by the soil ecosystem (Fanin et al., 2018). These communities are influential in processes such as the turnover of soil organic matter (SOM), soil carbon (C) sequestration, and water acquisition (Xiao and Veste, 2017). Essentially, the presence of abundant and diverse soil microorganisms, particularly in biocrusts, can greatly benefit various soil processes, especially those related to C and nitrogen (N) acquisition (Bhattacharyya and Furtak, 2022). During nutrient deficiencies, microorganisms obtain nutrients by increasing secretion of extracellular enzymes that decompose SOM (Cui et al., 2018). For example, in N-limited regions, it has been shown that soil organic N mineralization is related to soil extracellular N-acquisition enzymes (Xiao et al., 2021). These interactions between soil microbes and soil enzymes are essential for understanding nutrient limitations in land restoration in degraded areas (Liu et al., 2023).

Assembly processes of microbial communities play crucial roles in determining the rate and efficiency of microbial growth (Anthony et al., 2020). Community assembly arises from the interaction of deterministic factors such as heterogeneous selection, homogenous selection, and stochastic processes such as dispersion limitations and homogeneous dispersal (Xue et al., 2018). Assembly processes drive ecosystem functions (Knelman and Nemergut, 2014); they are mainly reflected in aspects such as climate regulation, nutrient cycle, and plant growth (Gao et al., 2020; Hartmann and Six, 2022). Stochastic processes could bring new species from the regional pool which carry traits affecting ecosystem functioning but are not present in the initial community. In this way, stochastic processes could enhance the effect of biodiversity on functions through sampling effects (Knelman and Nemergut, 2014). Meanwhile, microbial communities commonly display an inclined distribution of species abundance with a large proportion of rare taxa coexisting with a small number of abundant taxa (Lynch and Neufeld, 2015; Jia et al., 2018). Under low-salt-stress environments, the abundant taxa play an important role in stabilizing ecological networks. However, the role of rare taxa becomes more and more important when salt stress increases (Li et al., 2023). Furthermore, although rare taxa have a low abundance, they are highly diverse and have functional redundancy, such as nitrogen fixation, sulfur oxidation, and accelerating organic matter breakdown (Peter et al., 2011; Sauret et al., 2014; Hua et al., 2015). The community composition of rare taxa is more stable under the influence of climate change and other disturbances, such as copper stress, freeze-thaw, and mechanical disturbances (Pedrós-Alió, 2011). The role of abundant and rare microorganisms under moss crusts in karst areas remains poorly understood. The problem of nutrient limitation is more prominent in karst areas (Zhou et al., 2020). Researchers have highlighted the important role of abundant and rare taxa on nutrient acquisition. In the karst area, microbes under moss crusts show inconsistent assembly processes. Based on this background, understanding the contribution of abundant and rare taxa and to nutrient acquisition is essential concerning the restoration of karst areas. This knowledge can offer novel insights into the microorganisms under bryophytes in karst areas.

In the present study, we focused on three different moss habitats: (i) lithophytic moss crust of forest, (ii) autochthonal moss crusts of forest, and (iii) lithophytic moss crust of cliff. We used high-throughput amplicon sequencing based on 16S rRNA genes to evaluate the community structure and network stability of bacteria under moss crust. Enzyme stoichiometric analysis (EEA) was performed. We also studied the abundant and rare taxa assembly process and analyzed the links with soil physicochemical properties and nutrient acquisition. We hypothesized that (i) different bryophyte habitats lead to different soil properties and soil enzyme activities; (ii) the microbial response to nutrient restriction differs in the three habitats; and (iii) the assembly processes of abundant and rare microorganisms are inconsistent in different habitats. Bryophytes and microorganisms are closely linked, and this study also aimed to provide recommendations for the restoration of karst areas in Southwest China using bryophytes combined with associated microbes.

## 2 Materials and methods

### 2.1 Study sites and soil sampling

Soil samples were collected from Guiyang and Anshun, Guizhou Province, China (94°37′9′′#x2013;103°31′9′′ E, 36°56′9′′–40°34′9′′ N). In total, 36 soil samples under bryophytes were collected from three different sites (site A, 11 samples from lithophytic moss crust under the forest; site B, 17 samples from autochthonal moss crusts under the forest; site C, 8 samples from lithophytic moss crust of cliff). The study area is situated in a warm and subtropical region characterized by a humid temperate landscape with a continental monsoon pattern. The annual average rainfall is 1,100 mm and the mean air temperature ranges from 15.3 to 19.8°C. There is very little soil under the lithophytic moss crust. We collected the soil using the five-point sampling method to be mixed together as a sample; samples from the same type of moss habitat were replications. There were 11 replications in site A, 17 replications in site B, and 8 replications in site C. The specific sampling method is as follows: for lithophytic moss crust, we used a sterile blade to shovel the moss crust tightly against the rock wall; then, we used a sterile brush to sweep the roots of the moss crust and collect the soil. For autochthonal moss crust, we randomly selected some 1 m × 1 m plots in the study area. The nearest distance between sampling points in the sample field was approximately 10 m, and the sampling depth of soil samples under the crust was 0–2 cm. The soil samples were sieved to a particle size of 2 mm eliminating any discernible roots or rock fragments. The soil samples were separated into three portions: the first portion was kept at a temperature of −80°C to extract microbial DNA from the soil; we put the second portion at a temperature of 4°C to measure the activity of soil enzymes within a week; and the third portion was dried in air for analysis physical and chemical properties of the soil.

### 2.2 Determination of soil physical and chemical properties

Soil pH and electrical conductivity (EC) were measured using a pH-EC meter in the soil: water (1:5) extraction solution. Soil moisture content was determined gravimetrically. Soil samples were extracted with 2 M KCl and ammonium and nitrate nitrogen (NH_4_^+^-N and NO_3_^–^-N) were determined using an automatic flow injection analyzer (AutoAnalyzer-AA3, Sea Analytics, Norderstedt, Germany). Soil organic carbon (SOC) content was determined by the external heating method of K_2_Cr_2_O_7_ (Rayment and Lyons, 2011). Additionally, a Hanon Kjeltec 9840 analyzer (K9840, Hanon, CHN) was used to identify soil total nitrogen (TN) while a microplate reader (Infinite M200PRO, Tecan, CH) was used to measure the total phosphorus (TP) in the soil. We used a 0.5 M NaHCO_3_ solution and a microplate analyzer (Infinite M200PRO, Tecan, CH) to assess the available phosphorus (AP) for plants. Neutral ammonium acetate (1 M) was used to extract the soil available potassium (AK), and a flame spectrophotometer (FP6450, INESA, CHN) was used to measure the amount of AK.

### 2.3 Analysis of soil extracellular enzyme activity and the stoichiometry of extracellular enzymes

Quantification was made for the soil enzymes that are involved in carbon acquisition including α-1,4-glucosidase (AG), β-1,4-glucosidase (BG), xylosidase (XS), and β-D-cellobiohydrolase (CB); nitrogen acquisition enzymes including leucine aminopeptidase (LAP), and β-N-acetylglucosaminidase (NAG); phosphorus acquisition enzymes such as alkaline phosphatase (AP). The enzyme activities were evaluated using the microplate method described by Ai et al. (2012). All enzyme activities were standardized using *Z*-score to visually evaluate the differences between the three sites. We used Eq. 1 to calculate the *Z*-score:


(1) Z−s⁢c⁢o⁢r⁢e=x−μ/σ

where *x*, μ, and σ represent individual activity, average activity, and standard deviation of activity, respectively.

Two methods were used to investigate the microbial limitations. The first method was generating a scatter plot of the eco-enzymatic stoichiometry. The *x*-axis was determined by (LAP + NAG) / AP while the *y*-axis was determined by BG / (LAP + NAG). This strategy was based on the guidelines provided by Sinsabaugh et al. (2009). In this plot, four distinct categories of resource limitations were observed, as determined by deviations from the expected enzyme ratio of C:N (1:1) or N:P (1:1) as presented by Sinsabaugh et al. (2009). The soil enzyme activity ratio were calculated using Eqs 2–4.


(2) S⁢o⁢i⁢l⁢e⁢n⁢z⁢y⁢m⁢e⁢C:N⁢r⁢a⁢t⁢i⁢o=L⁢n⁢(B⁢G)/L⁢n⁢(L⁢A⁢P+N⁢A⁢G)

(3) S⁢o⁢i⁢l⁢e⁢n⁢z⁢y⁢m⁢e⁢C:P⁢r⁢a⁢t⁢i⁢o=L⁢n⁢(B⁢G)/L⁢n⁢(A⁢P)

(4) S⁢o⁢i⁢l⁢e⁢n⁢z⁢y⁢m⁢e⁢N:P⁢r⁢a⁢t⁢i⁢o=L⁢n⁢(L⁢A⁢P+N⁢A⁢G)/L⁢n⁢(A⁢P)

The second method was calculating the lengths and angles of the vectors for enzymatic activity to quantify the microbial nutrient limitation. The vector length indicates the relative microbial C limitation; the larger the vector length greater the relative microbial C limitation degree (Ma et al., 2021). The vector angle represents the soil relative microbial N (or P) limitation degree. Vector angles <45° indicate microbial N limitation, while vector angles >45° indicate microbial P limitation. The vector length and angle were calculated using Eqs 5–8.


(5) X=(B⁢G+C⁢B⁢H)/(B⁢G+C⁢B+A⁢P)

(6) Y=(B⁢G+C⁢B⁢H)/(B⁢G+C⁢B+N⁢A⁢G+L⁢A⁢P)

(7) $$V\,e\,c\,t\,o\,r\,l\,e\,n\,g\,t\,h=\sqrt{X^{2}+Y^{2}}$$

(8) V⁢e⁢c⁢t⁢o⁢r⁢a⁢n⁢g⁢l⁢e=D⁢e⁢g⁢r⁢e⁢e⁢(A⁢T⁢A⁢N⁢2⁢(X,Y))

### 2.4 DNA extraction and sequencing data processing

DNA Isolation Kit, which is produced by MP Biomedicals in Switzerland, was used to extract DNA from 0.5 g of fresh soil samples. The extraction process followed the manufacturer’s recommendations. The extracted DNA was assessed for its quality and quantity using a Nanodrop ND-2000 UV-vis spectrophotometer produced by Nanodrop Technologies in Wilmington, DE, USA. Primers 515F (5′-GTGCCAGCMGCCGCGGTAA-3′) and 806R (5′-GGACTACHVGGGTWTCTAAT-3′) were used to enhance the bacterial 16S rRNA gene V4 hypervariable region (Dong et al., 2022). PCR reactions were performed in triplicate (S1000 apparatus, Bio-Rad Laboratory, Hercules, CA, USA). The PCR conditions were 5 min at 94°C followed by 30 cycles of 94°C for 45 s, annealing at 54°C for 45 s, 72°C for 1 min followed by a final extension step of 10 min at 72°C (Katiraei et al., 2022). PCR products were purified using the Qiagen Gel Extraction Kit produced by Qiagen in Germany, following the instructions provided by the manufacturer. In the end, the library was sequenced using an Illumina Nova6000 platform, which produced paired end reads of 250 base pairs. The sequencing procedure was conducted by Guangdong Magigene Biotechnology Co., Ltd., situated in Guangzhou, China.

### 2.5 Sequencing data processing

A series of standard processing steps, including demultiplexing, sample inference, read merging, quality filtering, and chimeric elimination, were applied to the raw FASTQ data. The DADA2 pipeline (version 1.20.0) was used to carry out these procedures. After that, an amplicon sequence variant (ASV) microbiological profile was generated (Deissová et al., 2023), ASVs with 100% sequence identity, which are more dependable and can be replicated, were employed to represent microbial taxonomic units. The Ribosomal Database Project (RDP) Classifier was utilized to determine the taxonomic details of every ASV,^1^ with an 80% level of confidence (Matheri et al., 2023).

### 2.6 Statistics analysis

Depending on their relative abundance and/or frequency, the microbial ASVs were divided into two groups: abundant and rare taxa. Rare taxa were defined as those with an average relative abundance of <0.1%, whereas an average relative abundance of >1% was classified as abundant taxa (Galand et al., 2009). Based on the copiotrophic-oligotrophic framework and additional documentation, those annotated identified phyla into copiotrophic and oligotrophic taxa (Fierer et al., 2007; Li H. et al., 2021; Li J. et al., 2021). To calculate the microbial copiotroph:oligotroph ratios, the relative abundance of known copiotrophic and oligotrophic members were summed, respectively (Ma et al., 2023).

Also, we evaluated the α-diversity of the microbial community using the vegan package in R version 4.1.2. We employed canonical principal coordinates analysis (PCoA) to investigate the bacterial community’s pattern (Cui et al., 2019). All networks were constructed based on Pearson correlations of log-transformed ASV abundances, followed by an RMT-based approach that determines the correlation cut-off threshold automatically (Yuan et al., 2021). The random forest tests in “RandomForest” package were used to forecast the significant factors (Chen et al., 2021). Additionally, the “rfPermute” package in R was used to analyze each predictor’s significance (Jiao et al., 2018). To assess the ecological processes taking place in microbial communities, we calculated β Nearest Taxon Index (βNTI) (Stegen et al., 2012). Through the integration of —βNTI— (2) and —RCbray— (0.95), we successfully elucidated underlying mechanisms governing community assembly processes. These mechanisms encompass heterogeneous selection, homogeneous selection, dispersal limitation, homogenous dispersal, and undominated processes (Zhou and Ning, 2017). Mantel tests comparing βNTI values with the Euclidean distance matrixes of physicochemical parameters were then performed in the “vegan” R package to explore the major factors influencing the assembly of abundant and rare taxa, and the relationship among soil enzyme activity ratio and network complexity standardized by *Z*-scores. The direct and indirect effects between soil physical and chemical properties, assembly processes of abundant and rare microbial communities, microbial diversity, microbial complexity, and nutrient acquisition were identified using structural equation models (SEMs) created with the “lavaan” package in R software.

## 3 Results

### 3.1 Enzyme activity and soil nutrient limitation in different habitats

**FIGURE 1:**
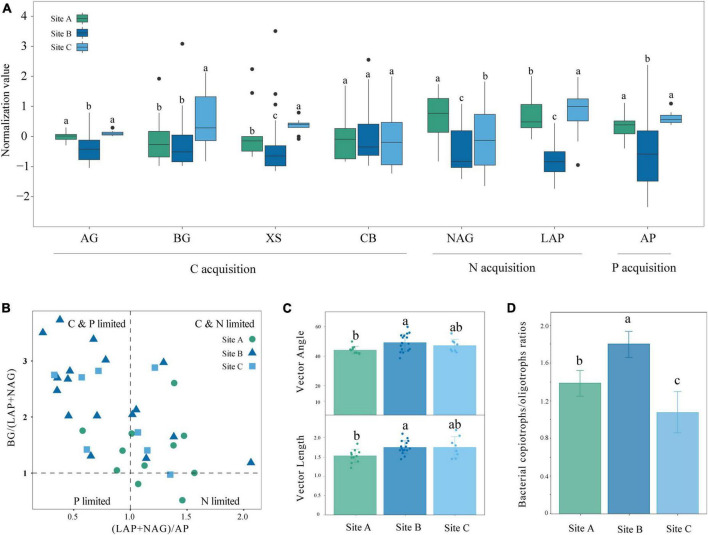
Enzyme activity and soil nutrient limitation in different moss biocrusts habitats: **(A)** soil enzyme activity of carbon, nitrogen, and phosphorus at three sites, **(B)** scatter plots of soil enzymatic stoichiometry for studied sites, **(C)** the vector length and vector angle of the studied sites, and **(D)** microbial copiotrophs and oligotrophic ratios at different sites. Different lowercase letters indicate significant differences among the three sites (*P* < 0.05). Different letters indicate statistically significant differences (one-way ANOVA, α = 0.05). AG, α-glucosidase; BG, β-glucosidase; XS, xylosidase; CB, β-D-cellobiohydrolase; NAG, N-acetyl-β-D-glucosidase; LAP, leucine aminopeptidase; AP, alkaline phosphatase.

The availability of SOC, NH_4_^+^-N, NO_3_^–^-N, and A-P in site B was higher, but the enzyme activities were lower compared with sites A and C (Table 1 and Figure 1A). The activity of BG, XS, NAG, and LAP showed significant differences in sites A and C (*P* < 0.05) (Figure 1A). The scatter plot of eco-enzymatic stoichiometry reveals that most microorganisms were limited by C&N and C&P (Figure 1B). Most microorganisms of the site A were limited by C&N, the microorganisms of sites B and C were susceptible to be limited by C&P (Figure 1B). Vector analysis showed that the site A had the lowest vector length among the three sites representing microbial C limitation. The vector angle at site A was below 45° whereas the vector angles at the other sites were above 45° (Figure 1C). Angles <45° are perceived as more constrained by N rather than P while the converse interpretation applies to angles >45°. This is consistent with the low content of SOC, NH_4_^+^-N, and NO_3_^–^-N in site A (Table 1). The soil microorganisms at site B were predominantly characterized by the r-strategy species, as indicated by the largest copiotroph/oligotroph ratio (Figure 1D). The high ratio reflected a more nutrient-rich environment.

**TABLE 1:** The physicochemical properties of soil under bryophytes in different sites.

Physico-chemical properties	Site A	Site B	Site C
pH	7.74 ± 0.3a	7 ± 1.04b	7.98 ± 0.05a
EC (μS/cm)	153.71 ± 42.69b	133.91 ± 67.84b	271.63 ± 27.92a
SWC (%)	8.54 ± 3.22b	21.28 ± 6.06a	5.85 ± 1.24b
SOC (g/kg)	41.36 ± 17.08b	44.19 ± 27.23b	64.94 ± 13.87a
TN (g/kg)	4.16 ± 1.72a	3.59 ± 2.14a	5.09 ± 1.17a
NH_4_^+^-N (mg/kg)	38.43 ± 13.12a	47.08 ± 20.35a	40.38 ± 14.18a
NO_3_^–^-N (mg/kg)	13.64 ± 10.06a	17.35 ± 9.89a	18.47 ± 9.97a
TP (g/kg)	0.99 ± 0.28a	0.58 ± 0.38b	0.66 ± 0.17b
A-P (mg/kg)	28.19 ± 16.33a	67.72 ± 103.04a	49.88 ± 19.6a
AK (mg/kg)	253.56 ± 51.49b	197.18 ± 61.61c	332.91 ± 47.44a

All values are reported as “mean @ standard deviation” based on measurement results for samples. The statistical differences in physicochemical properties within a row are indicated by different letters (one-way ANOVA, α = 0.05). pH, potential of hydrogen; EC, electric conductivity; SWC, soil water content; SOC, soil organic carbon; TN, soil total nitrogen; NH_4_^+^-N, soil ammonium; NO_3_^–^-N, soil nitrate; TP, soil total phosphorus; A-P, soil available phosphorus; AK, soil available potassium.

### 3.2 Diversity and co-occurrence network of soil microbial communities

**FIGURE 2:**
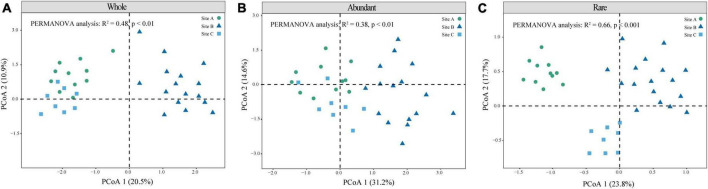
Microbial community structure of different moss crust habitats. Principal coordinate analysis (PCoA) of the whole **(A)**, abundant **(B)**, and rare **(C)** bacteria communities is given based on Bray–Curtis distances.

β-Diversity reflects differences in species composition between the three sites. The microbial community structures can be observed according to the principal coordinates analysis (PCoA), and permutational analysis of variance by Adonis. The results showed significance for the whole (*R*^2^ = 0.48, *P* < 0.01), abundant (*R*^2^ = 0.48, *P* < 0.01), and rare (*R*^2^ = 0.66, *P* < 0.001) communities (Figures 2A–C). While the structure of the rare community was obviously differentiated in all three sites (Figure 2C). The Shannon index of site B was significantly higher than that of the other two sites (Table 2) (*P* < 0.05). The α-diversity of rare communities was significantly different in the three sites (*P* < 0.05); however, there was no significant difference between the site C and the other two sites in abundant communities (Table 2). We further analyzed the correlation between the Shannon index of abundant and rare taxa and soil C, N, and P and their stoichiometry (Supplementary Figures 1, 2) and observed a significant correlation between rare taxa diversity and soil C, N, and P nutrients (Supplementary Figures 1, 2). For example, the diversity of rare taxa was negatively correlated with SOC (*R*^2^ = −0.38, *P* = 0.021), TN (*R*^2^ = −0.52, *P* = 0.0012), and TP (*R*^2^ = −0.4, *P* = 0.016). However, the diversity of abundant taxa had no significant correlations with soil C, N, and P nutrients. Further, we analyzed the relationship between the dominant phyla of rare microorganisms and soil nutrients (Supplementary Figure 3), most of which showed negative correlation. There were significant negatively correlations between TN and *Acidobacteriota*, *Actinobacteriota*, *Bacteroidota*, and *Desulfobacterota* (*P* < 0.05), and significant positively correlations were observed between C:N ratio and *Bacteroidota* and *Chloroflexi* (*P* < 0.05). These bacterial groups play an important role in soil C, N, and P nutrient cycling.

**TABLE 2:** The α-diversity (Shannon-index) in three sites.

Bacterial community	Site A	Site B	Site C
Whole	4.23 ± 0.39b	4.56 ± 0.62a	4.42 ± 0.59b
Abundant	3.13 ± 0.23b	3.56 ± 0.48a	3.42 ± 0.19ab
Rare	3.89 ± 0.46b	4.26 ± 0.72a	3.42 ± 0.38c

All values are reported as “mean ± standard deviation” based on measurement results for samples. The statistical differences in Shannon-index within a row are indicated by different letters (one-way ANOVA, α = 0.05).

**FIGURE 3:**
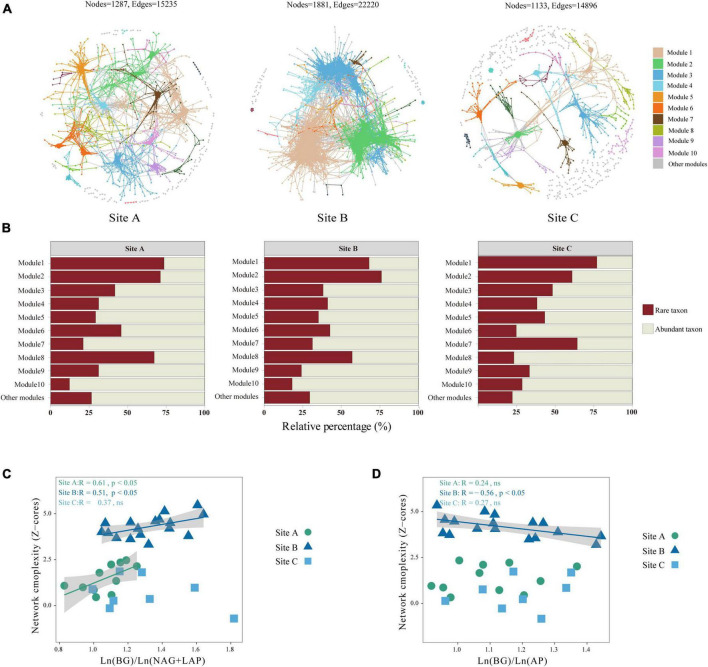
Co-occurrence pattern of bacteria in three sites: **(A)** the co-occurrence patterns among ASVs were revealed by network analysis. Large modules with ≥5 nodes are shown in different colors, and smaller modules are shown in gray. Details of network topological attributes are listed in Table 3: **(B)** ASVs abundance ratios of abundant and rare microorganisms in different modules; **(C)** relationships between network complexity standardized by *Z*-scores and Ln (BG)/Ln (NAG + LAP); and **(D)** relationships between network complexity standardized by *Z*-scores and Ln (BG)/Ln (AP). Different lowercase letters indicate significant differences among the three sites (*P* < 0.05). ns, not significant.

Most network nodes and edges were available in site B, resulting in highly clustered microbial network modules. Site C has the simplest network structure (Figure 3A). We used the network topological parameters of node and edge numbers, average degree, diameter, average clustering coefficient, and relative modularity to assess soil microbial network complexity, with higher topological properties representing greater network complexity. In the three sites, the network topology parameters of the site B were higher than those of the other two sites (Table 3). The key modules of the microbial co-occurrence network (major microbial clusters, modules 1 and 2) in the three habitats were dominated by rare taxa (Figure 3B). Our results showed that there was a significant positive correlation between Ln (BG)/Ln (NAG + LAP) and network complexity in sites A and B (*P* < 0.05) (Figure 3C). This indicates that the microbial network complexity increased as N limitation decreased in sites A and B. Further, there was a significant negative correlation between Ln (BG)/Ln (AP) and network complexity in site B (*P* < 0.05) (Figure 3D). This indicated that the complexity of microbial network was positively related to the level of P limitation in the site B. There was no significant relationship between network complexity and enzyme activity ratio in site C (Figures 3C, D).

**TABLE 3:** Topological properties of bacterial networks of three sites.

Topological properties	Site A	Site B	Site C
Node	1,287	1,881	1,133
Edges	15,235	22,220	14,896
Average degree	1.64	1.95	1.14
Diameter	10.46	13.54	9.57
Average clustering coefficient	0.38	0.45	0.26
Relative modularity	0.23	0.37	0.08

### 3.3 Assembly process and driving factors of abundant and rare taxa

**FIGURE 4:**
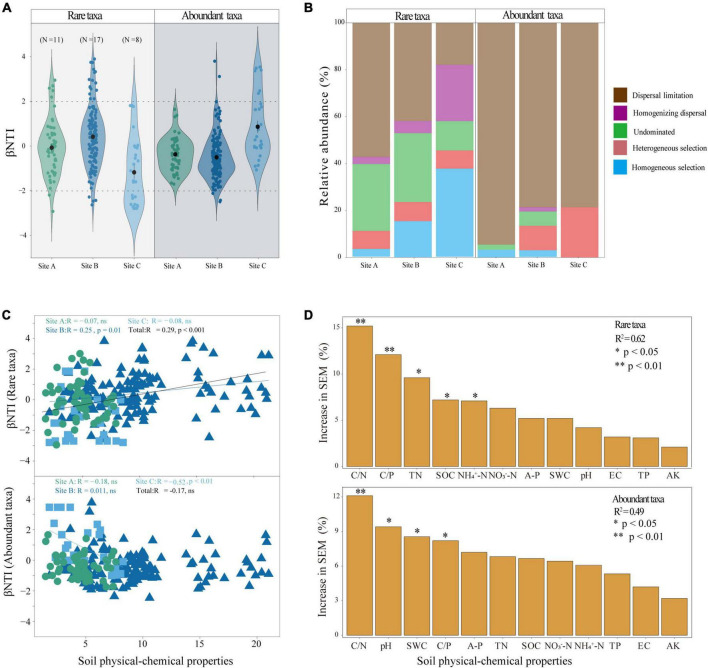
Assembly process and driving factors of abundant and rare taxa: **(A)** beta nearest taxon index (βNTI) value under different habitats. The horizontal dashed lines indicate the βNTI values of –2 and 2; **(B)** relative contribution of each ecological process to community assembly; **(C)** relationships between βNTI of the rare and abundant bacterial taxa and divergences of physical-chemical properties standardized; and **(D)** the random forest model identifies the major contributors to assembly processes of the abundant and rare taxa. ns, not significant. Asterisks denote significance levels. SWC, soil water content; EC, soil electric conductivity; TN, total nitrogen; AK, available potassium; A-P, available phosphorus; TP, total phosphorus; pH, potential of hydrogen; NO_3_^–^-N, nitrate nitrogen; NH_4_^+^-N, ammonium nitrogen; SOC, soil organic carbon; C/N, the ratio of soil organic carbon and total nitrogen; C/P, the ratio of soil organic carbon and total phosphorus.

In the three different habitats, most of the beta-nearest taxon index (βNTI) values of abundant taxa and rare taxa were between −2 and 2 indicating that the assembly process of abundant and rare taxa was dominated by stochastic processes (Figure 4A). However, the dispersal limitation of stochastic processes dominated the assembly of abundant taxa (Figure 4B). We observed that the assembly process of rare taxa was significantly (*P* < 0.001) correlated with the changes in soil physical and chemical properties (Figure 4C). To identify the potential main contributors to the assembly processes of abundant and rare taxa, we applied random forest analysis which demonstrated that C/N, C/P, TN, SOC, and NH_4_^+^-N were the significant impact factors in determining the rare taxa assembly process. C/N and C/P were significant factors (*P* < 0.01) (Figure 4D). Overall, C/N, pH, SWC, and C/P were the significantly impacting factors in determining the abundant taxa assembly process, and among them, C/N was significant (*P* < 0.01) (Figure 4D).

### 3.4 Microbial nutrient acquisition potential and driving factors

**FIGURE 5:**
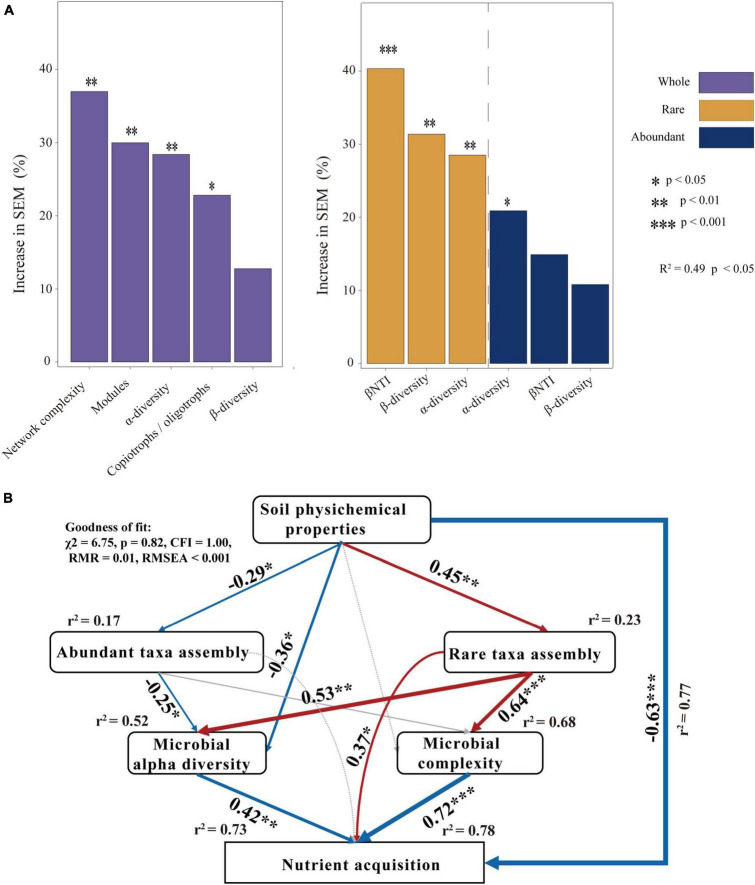
Analysis of factors affecting nutrient acquisition: **(A)** the random-forest model predicts the main factors for nutrient acquisition of bacterial communities and **(B)** structural equation model (SEM) revealing the direct and indirect effects of abundant taxon and rare taxon on nutrient acquisition. The numbers on the arrows are the path coefficients and are indicative of the standardized effect size of the relationship. Arrows in red and blue indicate positive and negative effects, respectively. *R*^2^ means the proportion of variance explained.

To explore the microbial factors that affect nutrient acquisition, we applied a random forest model. The random forest analysis revealed that network complexity, network modules α-diversity, and the ratio of copiotrophs and oligotrophs significantly affected nutrient acquisition in the whole microbial community. βNTI, β-diversity, and α-diversity significantly affected the nutrient acquisition of the rare microbial community. In the abundant microbial community, α-diversity significantly affected nutrient acquisition (Figure 5A). We further employed SEMs to explore the interaction influence between microbial factors by combining the physical and chemical properties of soil. The findings demonstrated that soil physicochemical properties had a significant effect on the assembly process of rare taxa leading to an increase in the complexity of the microbial network and diversity of community composition, and further stimulated nutrient acquisition (Figure 5B).

## 4 Discussion

### 4.1 Nutrient limitation on different bryophyte habitats

The availability of SOC, NH_4_^+^-N, NO_3_^–^-N, and A-P in sites A and C were lower but the enzyme activities were higher than those of site B (Tables 1, 4 and Figure 1A). This confirms that when the nutrient availability of the soil is low, microbes secrete more enzymes to meet the demand for nutrients (Sinsabaugh et al., 2002; Fierer et al., 2009; Wallenius et al., 2011; Xu et al., 2017). Significant differences were observed between site A and site C in the activity of BG, XS, NAG, and LAP (*P* < 0.05) (Table 4 and Figure 1A). Although sites A and C were both lithophytic moss crust habitats, site A was situated under forests, while site C was on the cliff. The difference made site A susceptible to shade from forests and site C susceptible to direct sunlight, resulting in varying soil moisture and nutrient conditions at the two sites. Soil nutrients have a significant influence on enzyme activities (Meier et al., 2020; Li et al., 2022). The scatter plot with eco-enzymatic stoichiometry reveals that microbial limitations in C&P, and C&N were prevalent across all locations (Figure 1B). It has been suggested that microbial C limitation is widespread (Schimel, 2003). N and P are mainly released from the decomposition of SOM (Pan et al., 2016; Alewell et al., 2020). Therefore, P or N limitation often coexists with C limitation in the three sites. Compared with site C, site A was not susceptible to C&P limitation (Figures 1B, C). This may be due to the higher diversity of rare taxa in site A (Table 2). Rare taxa exhibit diverse functions (Sauret et al., 2014), including microorganisms that can solubilize phosphorus and accelerate the breakdown of organic matter.

**TABLE 4:** The soil enzyme activities in three sites.

Soil enzymes	Site A	Site B	Site C
AG (μmol/L)	14.47 ± 5.38a	12.41 ± 7.47b	18.14 ± 4.57a
BG (μmol/L)	49.18 ± 25.4ab	44.52 ± 27.55b	73.48 ± 32.85a
XS (U/g)	20.98 ± 11.57b	16.39 ± 13.8c	26.75 ± 3.76a
CB (U/g)	19.84 ± 13.5a	14.94 ± 9.12a	17.78 ± 13.51a
NAG (U/g)	27.75 ± 9.92a	16.76 ± 9.74c	21.43 ± 13.97b
LAP (U/g)	7.67 ± 2.44b	3.23 ± 1.84c	8.69 ± 2.97a
AP (g/kg)	40.19 ± 14.94a	34.48 ± 11.31b	47.72 ± 10.45a

All values are reported as “mean ± standard deviation” based on measurement results for samples. The statistical differences in soil enzyme activities within a row are indicated by different letters (one-way ANOVA, α = 0.05). AG, α-glucosidase; BG, β-glucosidase; XS, xylosidase; CB, β-D-cellobiohydrolase; NAG, N-acetyl-β-D-glucosidase; LAP, leucine aminopeptidase; AP, alkaline phosphatase.

Site B had the higher nutrient availability and highest microbial diversity (Tables 1, 2), yet was still susceptible to significant nutrient limitations (Figures 1B, C). This can be explained by the nutritional strategies of microorganisms. Nutritional strategies involve a basic trade-off between the rate of growth and the efficiency of resource utilization (Fierer et al., 2007; Ho et al., 2017), enabling us to establish a direct correlation between microbial performance and environmental conditions. The soil microorganisms at site B were predominantly characterized as r-strategists, as indicated by the largest copiotroph/oligotroph ratio (Figure 1D). The r-strategy species (copiotrophic species) have a fast growth rate and a rapid response to available C and nutrient inputs, typically flourishing in environments enriched in nutrients (Yang et al., 2022c). In contrast, k-strategy species (oligotrophic species) are slow-growing and more common in oligotrophic environments (Yang et al., 2022c). The high ratio of copiotrophs results in significant nutrient demand, contributing to higher levels of nutrient limitations (Giovannoni et al., 2014). This further explains nutrient limitations of C, N, and P in site B (Figures 1B, C).

### 4.2 Diversity and complexity of soil bacterial under bryophytes

Microbial diversity depends on the availability of resources (Zhu L. et al., 2023). Varying ecosystems exhibit distinct soil temperatures, moisture levels, pH levels, and nutrient content which can influence the diversity and composition of soil microorganisms (Bahram et al., 2018). Site B had higher available nutrient content, such as NH_4_^+^-N, NO_3_^–^-N, and A-P than the other two sites (Table 1). However, sites A and C were characterized by lithophytic moss environments that exhibited low nutrient levels and supported oligotrophic microorganisms. The copiotroph/oligotroph ratio also showed the nutrient availability in the three sites (Figure 1D). This further explains the effect of different bryophyte habitats on microbial diversity. In addition, the abundant and rare microbial diversity of the three sites also showed distinct differences, rare microbes show a higher diversity (Table 2). Qin et al. (2022) indicated that rare biosphere possesses higher taxonomic diversity. This depended on multiple ecological principles behind the assembly of microbial communities (Lynch and Neufeld, 2015). The tremendous diversity of the rare biosphere is subjected to more complicated ecological processes such as speciation, drift, and extinction (Mo et al., 2018). Abundant communities have higher niche breadth and hence show strong resistance and adaptability to different environments, and spread in different ecosystems (Yang et al., 2022b). This explains the lack of clear structure of abundant communities in the three sites (Figure 2B). This study analyzed the relationship between abundant and rare microbial α-diversity and soil C, N, and P nutrients. There is usually a positive correlation between microbial diversity and soil nutrients (Chen et al., 2020); however, we found a negative relationship between diversity and soil nutrients in rare taxa (Supplementary Figure 1), inconsistent with previous findings. This can be explained by the competition for scarce nutrients occurs between bacteria and dominant plants, in nutrient-limited karst ecosystems (Wang et al., 2020). Furthermore, lower microbial metabolic activity may lead to the negative relationship between microbial diversity and soil nutrients (Hu et al., 2021). The karst rocky desertification ecosystem may have more dormant or inactive microorganisms, which explains that the metabolic activities of microorganisms are not strong, resulting in a slow turnover of resources. In addition, the soil C:N ratio in this study was much lower than the normal value, hindering the metabolic activities of soil microorganisms (Liu et al., 2018; Hu et al., 2021). Therefore, the dominant phyla of rare taxa were positively correlated with C/N, but negatively correlated with TN (Supplementary Figure 3). According to a previous study, there was a difference in structure and co-occurrence networks of the soil bacteria between different habitats (Zhong et al., 2022). Site B exhibited the highest complexity and community stability of the microbial network (Figure 3A). The complexity and stability of microbial networks are related to the network structure. As listed in Table 3, the network topology parameter of site B was the highest among the three sites. A previous study has reported a positive relationship between network complexity and microbial diversity (Ma et al., 2020). The microbial diversity at site B was also the highest (Table 2). We further found that rare taxa were the main component of microbial network modules 1 and 2 in the three sites (Figure 3B). The results indicated that rare taxa may play crucial roles in maintaining the structure of the co-occurrence network. Rare taxa form complex interactions and relationships with other taxa to maintain the network structure (Zhu M. et al., 2023; He et al., 2024). Reduced N limitation or enhanced P limitation increased microbial network complexity in site B (Figures 3C, D). Microorganisms in site B were more susceptible to P limitation than N limitation (Figure 1B). In this condition, soil microbial communities of site B can adapt their strategies in response to resource availability. Microbial strategies enhance their complexity to maintain a steady state (Yang et al., 2022c).

### 4.3 Factors of affecting nutrient acquisition

Community assembly describes how different ecological processes shape the composition and structure of microbial communities (Ning et al., 2024). Studying the effects of environmental factors on the assembly of microbial communities is critical to understanding microbial biodiversity and ecological function (Kang et al., 2023). The present study found that the community assembly of both abundant and rare microbes was mostly influenced by stochastic processes. Further, it was noted that the assembly process of abundant taxa was dominated by dispersal limitations of stochastic processes (Figure 4B). More individuals of abundant taxa are likely to be involved in dispersal events but vulnerable to physical barriers, distance and other factors (Liu et al., 2015). Diverse assembly processes contribute to the rare taxa (Figure 4B). Rare taxa occupy narrower niches, are closer in phylogenetic clustering, and are more likely to be filtered and dispersed by diverse assembly processes (He J. et al., 2022). Diverse assembly processes can drive high diversity and versatility of rare taxa (Zhou and Ning, 2017). From this perspective, the rare taxa may be the main driving force for maintaining ecosystem functional stability and diversity of the soil microbial communities (Gobet et al., 2012; Hugoni et al., 2013; Alonso-Sáez et al., 2015). Determining the elements that lead to the occurrence of different processes in the formation of microbial communities is essential in the study of community ecology. Prior studies have demonstrated a correlation between assembly processes and soil attributes such as pH and NH_4_^+^-N (Jiang et al., 2019; Wan et al., 2021). We found that soil C/N and C/P together significantly affected the community assembly of both abundant and rare taxa (*P* < 0.05) (Figure 4D). Especially, our findings indicated that TN, SOC and NH_4_^+^-N significantly influenced the assembly processes of the rare taxon. The assembly processes of the abundant taxa were significantly influenced by soil pH and SWC (*P* < 0.05) (Figure 4D). Soil C, N, and P are essential nutrients in soil ecosystems, and the stoichiometric ratio of soil C, N, and P affected the assembly process of microorganisms by affecting microbial investment in nutrient acquisition (Li J. et al., 2021; Xu et al., 2022; Duan et al., 2023). Therefore, both abundant and rare microbes were affected by soil C/N and C/P. It was worth distinguishing that abundant taxa were more susceptible to the non-nutrient properties of soil, such as pH and SWC. Soil pH and SWC can affect the cellular homeostasis of microorganisms and thus affect the exchange with external substances. The versatility of rare microbes allows for their ability to withstand substantial variances and be less affected by changes in environmental pH and SWC (He Z. et al., 2022). In total, the SEM demonstrated that the physical and chemical properties of soil mainly significantly affected the community assembly of the rare taxa, indirectly affecting microbial diversity and complexity and finally nutrient acquisition (Figure 5B). This suggested that rare microbial taxa were major drivers of nutrient.

## 5 Conclusion

This study aimed to determine the response of microorganisms under moss crust to nutrient acquisition in karst area. There were nutrient limitations in the three habitats. Our study demonstrated that nutrient acquisition was mainly driven by microbial assembly, diversity, and complexity. The microbial diversity and complexity were higher in the autochthonal moss crust of forest compared to the lithophytic moss crust. The microbial responses were influenced by the specific habitats of bryophytes, especially differences in soil nutrients. To enhance the efficiency of ecological restoration initiatives in karst rocky desertification areas, it is imperative to understand the influence of soil microbial communities on ecosystem processes. Further, with a full understanding of local nutrient limitations, the practice of cultivating bryophytes alongside rare microorganisms serves as a significant approach to managing and mitigating the process of rocky desertification. By addressing these technological limitations, it is possible to enhance the effectiveness and precision of the restoration activities aimed at mitigating karst rocky desertification and land degradation.

## Data availability statement

The datasets presented in this study can be found in online repositories. The names of the repository/repositories and accession number(s) can be found in the article/Supplementary material.

## Author contributions

XD: Conceptualization, Investigation, Methodology, Writing – original draft. MC: Conceptualization, Formal analysis, Investigation, Methodology, Writing – original draft. QC: Formal analysis, Software, Writing – review & editing. KL: Data curation, Methodology, Writing – review & editing. JL: Formal analysis, Software, Writing – review & editing. YL: Investigation, Methodology, Writing – review & editing. YR: Data curation, Investigation, Writing – review & editing. TY: Software, Writing – review & editing. JZ: Funding acquisition, Writing – review & editing. SH: Supervision, Writing – review & editing. XP: Funding acquisition, Supervision, Visualization, Writing – review & editing.

## Footnotes

1
https://sourceforge.net/projects/rdp-classifier/
