# Supplementary material for: Rare microbial taxa as the major drivers of nutrient acquisition under moss biocrusts in karst area
Source: Front Microbiol. 2024 May 1;15:1384367. doi: 10.3389/fmicb.2024.1384367 (PMC11094542; doi:10.3389/fmicb.2024.1384367)
Supplement: Supplementary file 1 [file Presentation_1.pdf]

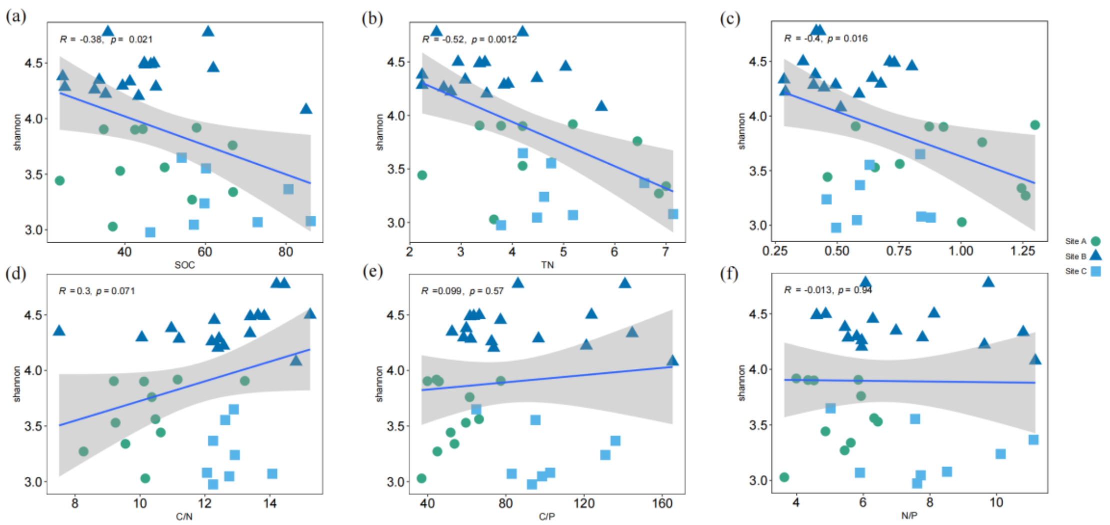

FIGURE S1 | Relationships between soil carbon, nitrogen and phosphorus nutrients attributes and the diversity of rare taxa. (a) Relationships between soil organic carbon and shannon index of rare taxa. (b) Relationships between soil total nitrogen and shannon index of rare taxa. (c) Relationships between soil total phosphorus and shannon index of rare taxa. (d) Relationships between ratio of soil organic carbon to total nitrogen and shannon index of rare taxa. (e) Relationships between ratio of soil organic carbon to total phosphorus and shannon index of rare taxa. (f) Relationships between ratio of soil total nitrogen to total phosphorus and shannon index of rare taxa.

Notes: The abbreviations are listed as follows. SOC, soil organic carbon; TN, total nitrogen; TP, total phosphorus; C/N: the ratio of soil organic carbon and total nitrogen; C/P: the ratio of soil organic carbon and total phosphorus; N/P: the ratio of soil total nitrogen and total phosphorus.

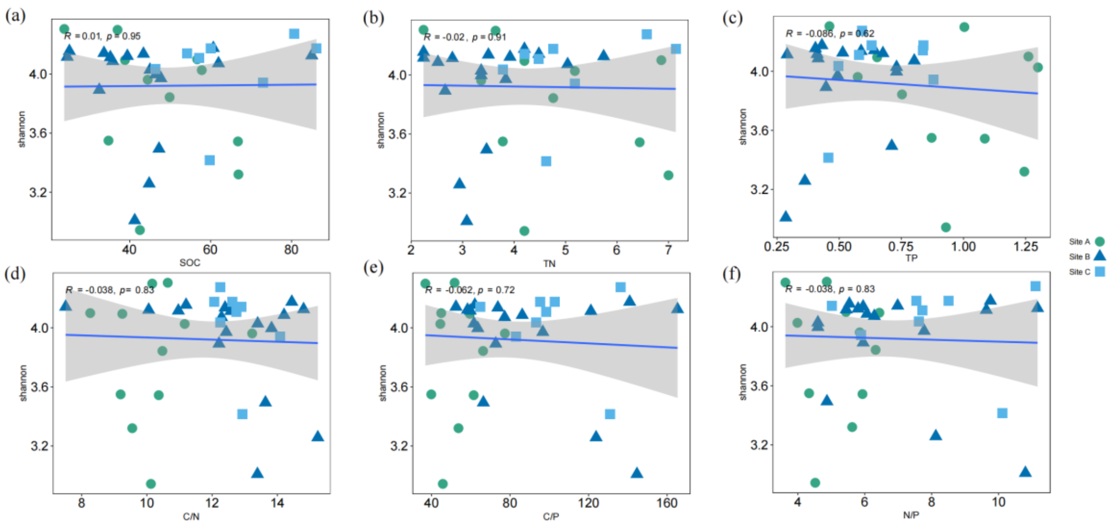

FIGURE S2 | Relationships between soil carbon, nitrogen and phosphorus nutrients attributes and the diversity of abundant taxa. (a) Relationships between soil organic carbon and shannon index of abundant taxa. (b) Relationships between soil total nitrogen and shannon index of

abundant taxa. (c) Relationships between soil total phosphorus and shannon index of abundant taxa. (d) Relationships between ratio of soil organic carbon to total nitrogen and shannon index of abundant taxa. (e) Relationships between ratio of soil organic carbon to total phosphorus and shannon index of abundant taxa. (f) Relationships between ratio of soil total nitrogen to total phosphorus and shannon index of abundant taxa.

Notes: The abbreviations are listed as follows. SOC, soil organic carbon; TN, total nitrogen; TP, total phosphorus; C/N: the ratio of soil organic carbon and total nitrogen; C/P: the ratio of soil organic carbon and total phosphorus; N/P: the ratio of soil total nitrogen and total phosphorus.

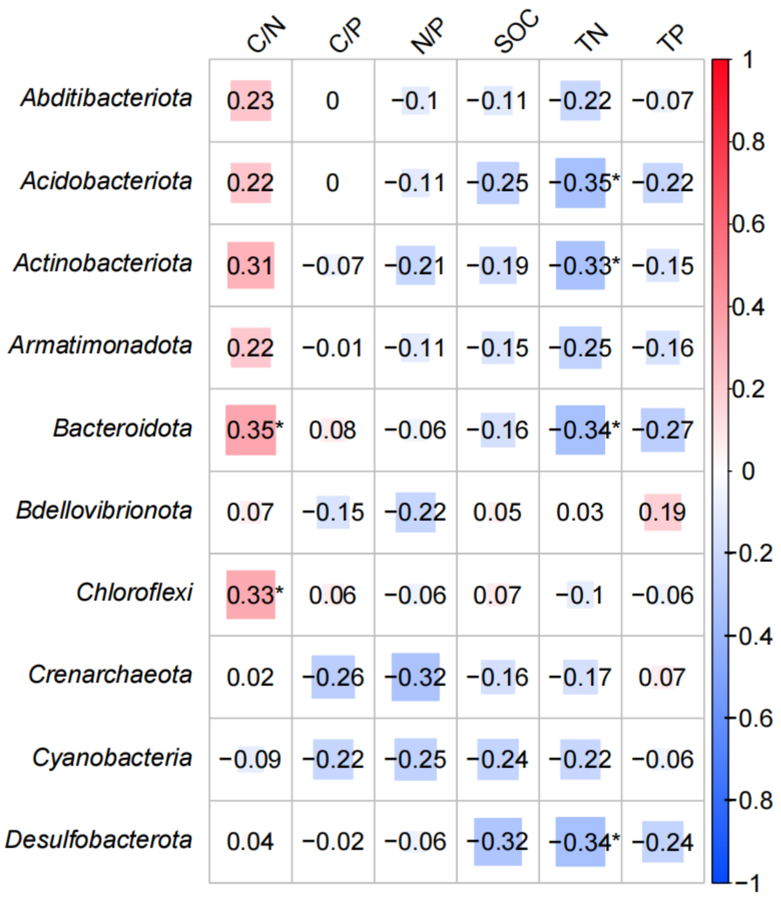

	C/N	C/P	N/P	SOC	TN	TP
Abditibacteriota	0.23	0	−0.1	−0.11	−0.22	−0.07
Acidobacteriota	0.22	0	−0.11	−0.25	−0.35*	−0.22
Actinobacteriota	0.31	−0.07	−0.21	−0.19	−0.33*	−0.15
Armatimonadota	0.22	−0.01	−0.11	−0.15	−0.25	−0.16
Bacteroidota	0.35*	0.08	−0.06	−0.16	−0.34*	−0.27
Bdellovibrionota	0.07	−0.15	−0.22	0.05	0.03	0.19
Chloroflexi	0.33*	0.06	−0.06	0.07	−0.1	−0.06
Crenarchaeota	0.02	−0.26	−0.32	−0.16	−0.17	0.07
Cyanobacteria	−0.09	−0.22	−0.25	−0.24	−0.22	−0.06
Desulfobacterota	0.04	−0.02	−0.06	−0.32	−0.34*	−0.24

FIGURE S3 | Analysis of correlation between the soil nutrients and dominant phylum of rare taxa

Notes: The abbreviations are listed as follows. SOC, soil organic carbon; TN, total nitrogen; TP, total phosphorus; C/N: the ratio of soil organic carbon and total nitrogen; C/P: the ratio of soil organic carbon and total phosphorus; N/P: the ratio of soil total nitrogen and total phosphorus.
